# *Lactobacillus casei* Shirota Supplementation Does Not Restore Gut Microbiota Composition and Gut Barrier in Metabolic Syndrome: A Randomized Pilot Study

**DOI:** 10.1371/journal.pone.0141399

**Published:** 2015-10-28

**Authors:** Vanessa Stadlbauer, Bettina Leber, Sandra Lemesch, Slave Trajanoski, Mina Bashir, Angela Horvath, Monika Tawdrous, Tatjana Stojakovic, Günter Fauler, Peter Fickert, Christoph Högenauer, Ingeborg Klymiuk, Philipp Stiegler, Manfred Lamprecht, Thomas R. Pieber, Norbert J. Tripolt, Harald Sourij

**Affiliations:** 1 Medical University of Graz, Division of Gastroenterology and Hepatology, Graz, Austria; 2 Medical University of Graz, Division of Transplantation Surgery, Graz, Austria; 3 Centre for Medical Research, Graz, Austria; 4 Medical University of Graz, Division of Endocrinology and Metabolism, Graz, Austria; 5 Medical University of Graz, Clinical Institute of Medical and Chemical Laboratory Diagnostics, Graz, Austria; 6 Medical University of Graz, Institute of Physiological Chemistry, Centre for Physiological Medicine, Graz, Austria; 7 Centre for Biomarker Research in Medicine (CBmed), Graz, Austria; The Chinese University of Hong Kong, HONG KONG

## Abstract

Metabolic syndrome is associated with disturbances in gut microbiota composition. We aimed to investigate the effect of *Lactobacillus casei* Shirota (*Lc*S) on gut microbiota composition, gut barrier integrity, intestinal inflammation and serum bile acid profile in metabolic syndrome. In a single-centre, prospective, randomised controlled pilot study, 28 subjects with metabolic syndrome received either *Lc*S for 12 weeks (n = 13) or no *Lc*S (n = 15). Data were compared to healthy controls (n = 16). Gut microbiota composition was characterised from stool using 454 pyrosequencing of 16S rRNA genes. Serum bile acids were quantified by tandem mass spectrometry. Zonulin and calprotectin were measured in serum and stool by ELISA. *Bacteroidetes/Firmicutes* ratio was significantly higher in healthy controls compared to metabolic syndrome but was not influenced by *Lc*S. *Lc*S supplementation led to enrichment of *Parabacteroides*. Zonulin and calprotectin were increased in metabolic syndrome stool samples but not influenced by *Lc*S supplementation. Serum bile acids were similar to controls and not influenced by *Lc*S supplementation. Metabolic syndrome is associated with a higher *Bacteroidetes/Firmicutes* ratio and gut barrier dysfunction but *Lc*S was not able to change this. *Lc*S administration was associated with subtle microbiota changes at genus level.

**Trial Registration:**

ClinicalTrials.gov NCT01182844

## Introduction

The obesity epidemic is a challenging threat to public health in the 21^st^ century. The World Health Organisation states that by 2014 approximately 1.9 billion adults are overweight (BMI 25–29.9 kg/m^2^), and more than 600 million are obese (BMI 30 kg/m^2^ or more). [1] Obesity and insulin resistance are major risk factors for the development of metabolic syndrome (MetS), type 2 diabetes mellitus (T2DM), and conditions such as cardiovascular morbidity and mortality. [2, 3]

The pathogenesis of obesity is multifactorial and is seen as interplay between individual phenotype and environmental factors. However, recent preclinical and clinical studies show an important influence of the intestinal microbiota on obesity and associated metabolic disorders (MetS, T2DM, metabolic liver diseases, cardiovascular diseases). [4] The cecum microbiota of obese mice was found to be significantly different with a higher prevalence of *Firmicutes* and a corresponding lower prevalence of *Bacteroidetes* (lower *Bacteroidetes/Firmicutes* ratio) in obese compared to lean mice. [5] This finding of altered gut microbiota was then confirmed and extended to humans. [6] However, others did not find this association in obesity or MetS. [7–10] Further studies have shown an association of gut microbiota changes with insulin resistance and diabetes. [11, 12] This suggests that the microbiota might be involved in the pathogenesis of obesity, insulin resistance and T2DM, possibly by having an impact on gut barrier integrity and inflammation. [4] Also, hydrophobic bile acids have been proposed as a novel mechanism for high fat diet induced gut barrier dysfunction. [13, 14] Probiotic interventions have been shown to be effective in modulating gut barrier integrity and gut microbiota in animals and thereby modulating chronic inflammation and metabolic disorders in animal models. [15, 16] Despite several mechanistic studies and encouraging results in animals [17, 18] interventional data on probiotics use in humans with MetS are rare. [19]

The aim of our study was to investigate the effect of *Lactobacillus casei* Shirota (*Lc*S) on gut microbiota composition, gut barrier integrity, intestinal inflammation and the serum bile acid profile in MetS.

## Patients and Methods

### Patients and Controls

The study was conducted according to the Declaration of Helsinki and all procedures involving human subjects were approved by the Ethics Committee of the Medical University of Graz (20–037 ex 08/09). The study was registered at ClinicalTrials.gov (NCT01182844). Due to an unexpected organizational delay in the registration process of the study the initial release of the protocol in ClinicalTrials.gov took place after the first patient was randomized. The authors confirm that they have not performed and are not conducting any other trials with this intervention (*Lactobacillus casei* Shirota). Written informed consent was obtained from all subjects. The primary and parts of the secondary outcomes were already published [20, 21]. The gut microbiome analysis was delayed due to lack of funding. The Ethics Committee of the Medical University was informed about the delay in analysis and about all changes in the study protocol. These changes were methodological changes since the methods for gut microbiome analysis and gut permeability analysis have advanced since the start of the study. Furthermore the analysis of bile acids has been added to the protocol and was approved by the Ethics Committee of the Medical University.

According to the modified NCEP-ATP-III-guidelines [22] patients with MetS were identified from the outpatient clinic at the Division of Endocrinology and Metabolism at the Medical University of Graz. Patients treated with antibiotics within the previous 7 days, with current anti-hyperglycemic treatment, any immunomodulatory therapy 1 month prior to study entry, concomitant use of pre-, pro-, or synbiotics, inflammatory bowel disease (Crohn`s disease, ulcerative colitis) or celiac disease or those with clinical signs of infectious diseases were excluded from participation. We performed a single-centre, permuted-block randomised controlled 12 weeks prospective intervention trial. Patients were randomised to receive either food supplementation with a milk drink containing *Lc*S (3 bottles a day, à 65ml, containing *Lc*S at a concentration of 10^8^/ml, Yakult light®, Yakult Austria, Vienna, Austria) for twelve weeks (n = 13, LcS group) or no intervention (n = 15, standard therapy group).

Patients were randomised with the “Randomizer®” software using permutated blocks (Institute for Medical Informatics, Statistics and Documentation, Medical University of Graz, Austria). All patients were advised to consume no other probiotic supplements during the study period and received a list of probiotic products available in Austria, which they had to avoid for the study period. Participants were also advised not to change their diet and physical activity pattern while being in the study. A food frequency questionnaire was used to confirm unchanged diet habits. [20] Subjects of the *Lc*S group were provided with a pack of the milk drink every two weeks. At these time points intervention adherence was assessed.

Stool and serum samples were collected at baseline and after 12 weeks. Stool samples from 16 healthy, lean controls without evidence of metabolic syndrome were used as a comparison for gut microbiota analysis and zonulin levels. For stool calprotectin established reference ranges from the kit description were used. Serum samples from 11 healthy, lean controls were used to compare bile acid profiles, zonulin, and calprotectin levels in serum.

### DNA-isolation, 454 library preparation and sequencing

Stool samples were immediately frozen and stored at -80°C until semi-automated DNA isolation. Approximately 175mg of stool was homogenized in MagnaLyser Green Bead tubes by using the MagnaLyser Instrument (Roche Diagnostics, Mannheim, Germany) according to manufacturer’s instructions. Total genomic DNA was isolated with the MagNA Pure LC DNA Isolation Kit III (Bacteria, Fungi) in a MagNA Pure LC 2.0 Instrument (Roche Diagnostics, Mannheim, Germany) according to manufacturer’s instructions. Enzyme cocktail II (Roche Diagnostics, Mannheim, Germany) with 100μg lysozyme (Karl Roth GmbH, Karlsruhe, Germany) per 100μl sample was used according to manufacturer’s instructions.

The 16S rRNA gene was amplified using FLX 454 one way read (Lib-L kit, Primer A, Primer B, Roche 454 Life Science, Branford, CT, USA) (S1 Table) fusion primers with the template specific sequence F27—AGAGTTTGATCCTGGCTCAG and R534—ATTACCGCGGCTGCTGGC targeting the V1-V3 hypervariable regions [23, 24] as described previously in Kump et al. 2013. [25]

### Zonulin and calprotectin

A ready-to-use solid-phase sandwich ELISA (Immundiagnostik AG, Bensheim, Germany) was used to detect zonulin (zonulin Serum or Stool ELISA) and calprotectin (PhiCal® calprotectin Serum or Stool ELISA) in serum and stool samples. The tests were performed according to the manufacturer’s instructions. For stool sampling the Stool Sample Application System (Immundiagnostik AG, Bensheim, Germany) was used according to the manufacturer’s manual.

### Bile acids

All bile acids (cholic acid, CA; deoxycholic acid, DCA; chenodeoxycholic acid, CDCA; lithocholic acid, LCA; ursodeoxycholic acid, UDCA) were assessed as unconjugated acids and as taurine and glycine conjugates using a tandem mass spectrometry method as described previously. [26] All sub-fractions of bile acids (free acids and their corresponding conjugates) were analysed by three different multiple-reaction monitoring experiments within one HPLC run. HPLC was performed on a reversed-phase (C18) column that used a methanol/water gradient for chromatographic solution of isobaric bile acids. Deuterated internal standards and correlation of peak area ratios in linear regression were used for quantification of all sub-fractions of bile acids.

#### Data analysis

Raw sequencing data generated on the Genome Sequencer FLX system were de-noised with Acacia 1.52. Afterwards reads were pre-processed with several quality parameters to trim primer and barcode sequences and filter low quality reads according to suggestions described in Huse et.al. [27]. Data analysis was performed in QIIME 1.7.0 [28] and included following steps: clustering of high quality reads into operational taxonomic units (OTUs) using UCLUST [29] and similarity of 0.97; taxonomy assignment of the OTUs with RDP classifier [30] and confidence score of 0.8 based on GreenGenes [31]16S rRNA database; representative sequence alignment with PyNAST [32]; detection and removal of chimeric sequences with ChimeraSlayer; [33] generating a phylogenetic tree with FastTree [34]. Additionally singletons (OTUs with one read present only in one sample) were removed to avoid overestimation of the sample richness and diversity. Finally, the resulting OTU table and accompanied phylogenetic tree were further used for calculation of alpha and beta diversity. Alpha diversity indices were determined with the R statistical programming language extended with Vegan and BiodiversityR community ecology packages whereas beta diversity was done in QIIME involving data rarefaction to the smallest sample size and selecting two distance measurements, weighted UniFrac and Bray-Curtis distance for the Principal Coordinates Analysis (PCoA) diagrams and between samples comparisons. Testing whether groups of the samples were significantly different was assessed with the non-parametric MANOVA method implemented in the Vegan package (Adonis) or with Student’s t-Test and ANOVA as implemented in R environment.

We searched for *Lactobacilli*, because sequencing only a fragment of the 16S rRNA gene does not allow us to determine species with a high confidence.

Searching for *Akkermansia* was done using taxonomic information in the OTU table.

All other statistical analyses were performed using SPSS 18.0 software (SPSS Inc, Chicago). The Mann-Whitney-U test or the unpaired student’s t-test were used for the comparison of differences between groups and the paired student’s t-test or the Wilcoxon signed-rank test for the before and after treatment measurements, as appropriate for normally and not-normally distributed variables, respectively. Differences with a p-value below 0.05 were considered statistically significant and for multiple tests we used Benjamini & Hochberg correction.

## Results

### Patients

**Fig 1:**
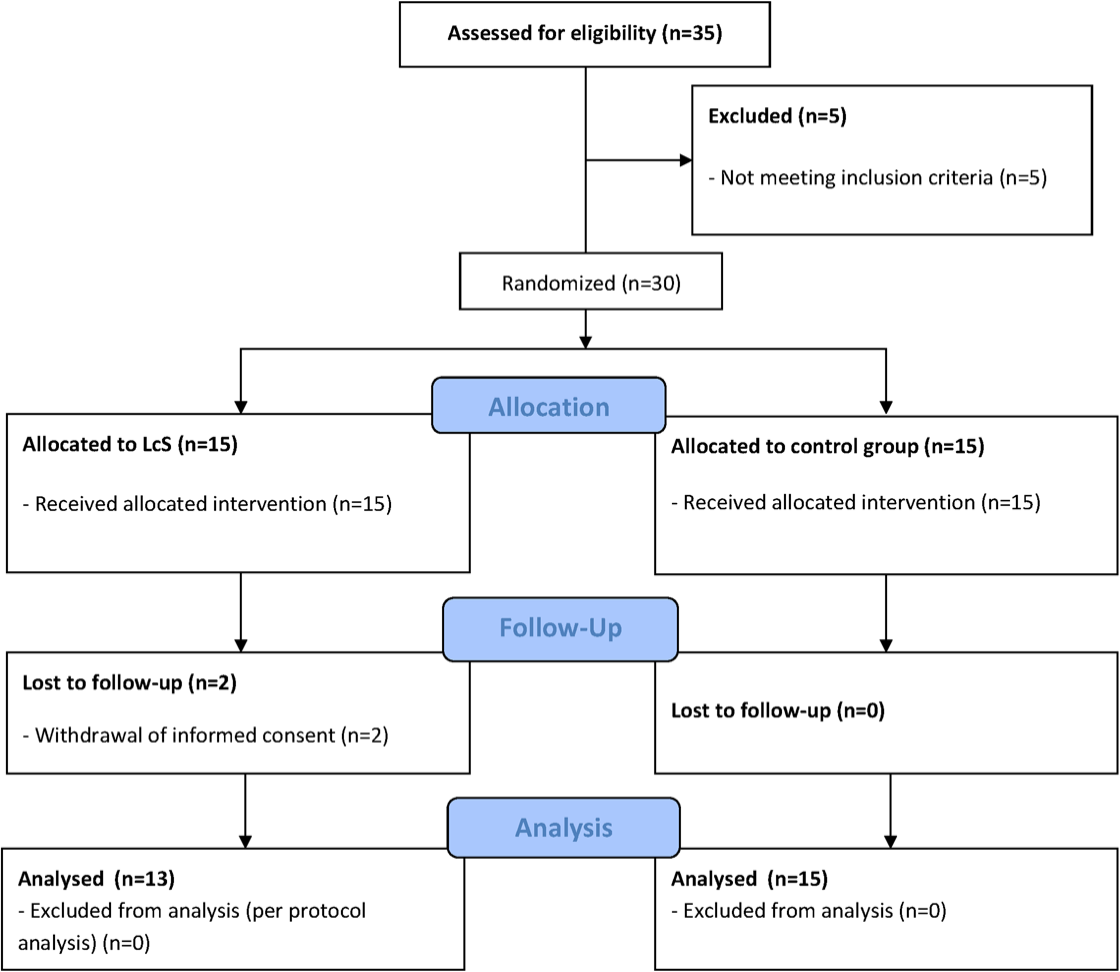
Flow diagram of the study progress.

Thirty-five subjects were screened for the study between January and August 2010; 30 patients were finally included, whereof 28 finished the study (2 dropped out due to withdrawal of informed consent). Five patients did not fulfill the inclusion criterion of fasting glucose above 100 mg/dl at the day of screening any more. Thirteen patients were randomized to the probiotic group and 15 to the standard therapy group (Fig 1). Baseline characteristics of patients with MetS and a healthy control group are shown in Table 1.

**Table 1:** Patient characteristics. Data are given as mean±SD.

	*Lc*S (n = 13)		Standard therapy (n = 15)		Healthy (n = 16)
	base	EOS	base	EOS	
Sex (female/male)	4/9	4/9	6/9	6/9	7/9
Age (years)	51±11	51±11	55±9	55±9	25±4***
Height (cm)	175±8	175±8	169±8	169±8	172±8
Weight (kg)	109±15	108±17	91±14**	91±15	69±11***
Blood pressure systolic (mmHg)	148±19	142±16	147±18	139±11	n.a.
Blood pressure diastolic (mmHg)	95±12	92±12	94±18	88±9	n.a.
Body mass index (kg/m^2^)	35±5	35±6	32±4*	32±4	23±3***
Waist circumference (cm)	113±12	112±12	106±8	106±9	76±8***
Total cholesterol (mmol/l)	220±69	219±59	209±43	211±34	4.9±1
High density lipoproteins (mmol/l)	43±17	40±16	47±19	42±12	1.6±0.3*
Low density lipoproteins (mmol/l)	128±49	132±45	119±27	126±29	2.7±0.9
Triglycerides (mmol/l)	214±169	202±123	170±106	159±66	1.2±0.5*

*p<0.05

**p<0.01

***p<0.001 compared to the other groups at baseline

n.a. not available; EOS: end of study; base: baseline; healthy: healthy controls.

### Gut microbiota

We generated 539,934 raw sequences with a mean length of 362bp. After de-noising and quality filtering, 390,021 reads (mean length 393bp) remained for downstream analysis. Chimeric sequences (18.5%) and singletons (2.71%, reads occurring only once in a single sample) were removed and not used for further analysis. The average number of reads per sample was 5,208 (SD 1,786; range 1,716–11,252). Rarefaction curves confirmed that sequencing effort was not sufficient to cover all rare taxa, but still the curve trends were approaching saturation, and did not show any specific group dependent characteristics.

#### Comparison between MetS patients and healthy controls

**Fig 2:**
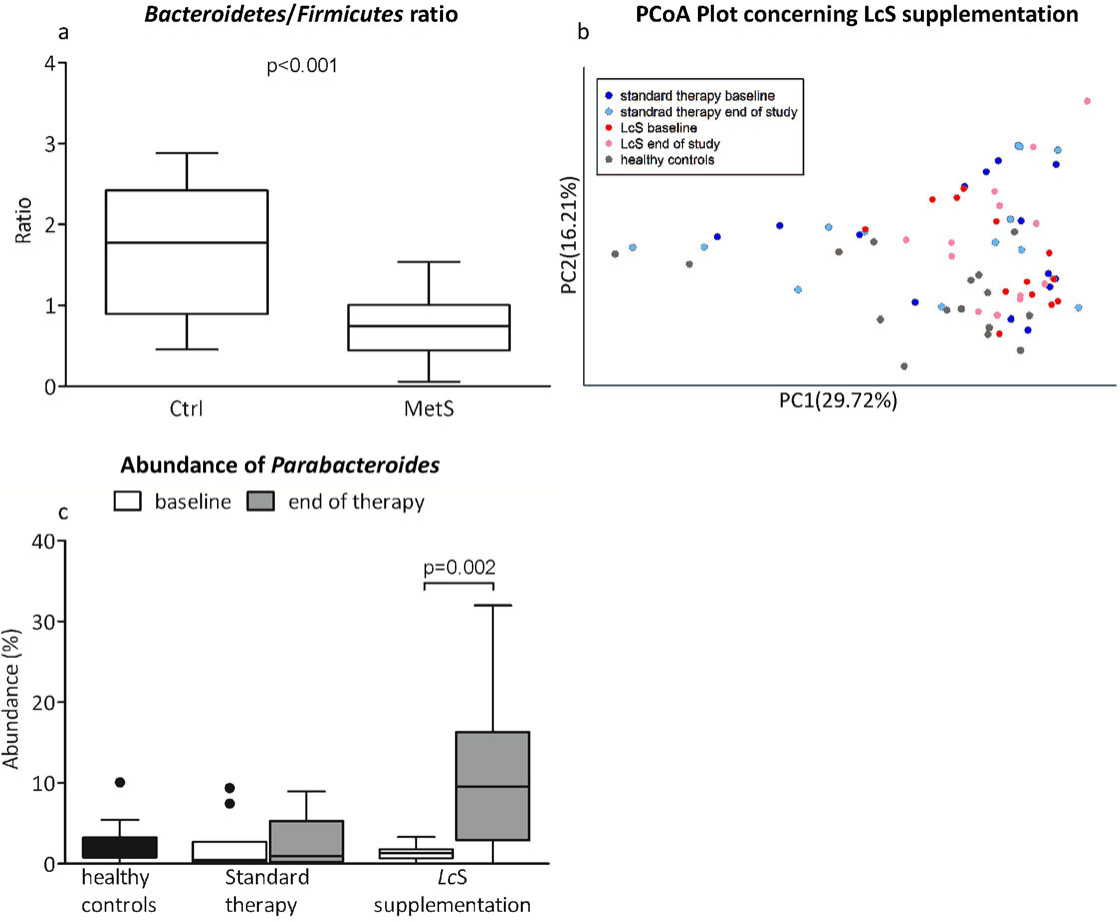
Gut microbiota composition in MetS patients and controls. *Bacteroidetes*/*Firmicutes* ratio (a) PCoA Plot (Weighted UniFrac, b) and abundance of *Parabacteroides* (c) concerning *Lc*S supplementation.

*Bacteroidetes/Firmicutes* ratio was significantly lower in patients with MetS (median: 0.75; quartiles: 0.44–1.00; p<0.0001) compared to healthy controls (median: 1.77; quartiles: 0.89–2.42; Fig 2A). We could recognize two groups of samples, where *Bacteroides* (68%) or *Prevotella* (15%) was most abundant as well as different other genera making the rest of the samples (17%). No statistical differences in the *Bacteroides* and *Prevotella* groups’ distribution between patients and controls were detected. No significant differences in UniFrac (p = 0.70) or Bray-Curtis (p = 0.48) distances between baseline and end of therapy were found. Evenness and diversity (Shannon and Simpson Index) of gut microbiota in MetS showed similar distribution compared to healthy controls (Table 2).

**Table 2:** Evenness and diversity of gut microbiota in MetS and healthy controls. Data are given as median (quartiles).

	MetS patients (n = 28)	Healthy controls (n = 16)	p-value
J-evenness	0.62 (0.56–0.66)	0.65 (0.59–0.66)	0.238
Shannon	3.8 (3.5–4.2)	3.9 (3.5–4.1)	0.839
Simpson	0.91 (0.88–0.95)	0.92 (0.87–0.95)	0.985

MetS: metabolic syndrome.

*Akkermansia*, a possibly relevant genus in the pathophysiology of MetS, was detectable in higher abundance in only one individual in the standard therapy group at baseline and at the end of the study (21.40% and 24.28%, respectively).

#### Effect of supplementation of *Lc*S in MetS

Variations (UniFrac distances) in microbiota composition found in the *Lc*S group between baseline and end of the study were similar to variations in the standard therapy group. Diversity of gut microbiota in MetS was not influenced by *Lc*S supplementation. We also could not detect any significant changes in microbiota between the *Lc*S and standard therapy group at any time point using Adonis multivariate analysis. Weighted UniFrac PCoA plots showed no clear separation of the groups between the two time points and in comparison with the healthy controls (Fig 2B).

By looking at the most abundant genera however (present with more than 1% in at least 50% of the samples) we found a significantly increased proportion of *Parabacteroides* at the end of the study when compared to baseline in the *Lc*S group (p = 0.002). (Fig 2C)

*Lactobacillus* genus was not detectable with our method.

### Gut wall integrity and inflammation

Zonulin and calprotectin were studied as markers of gut barrier disruption and intestinal inflammation. Serum levels of zonulin and calprotectin were not elevated in patients compared to healthy controls. However, zonulin and calprotectin were significantly higher in stool samples of patients compared to controls (p<0.001) or compared to the median of healthy controls from the kit description (p = 0.017). Both zonulin and calprotectin levels were not influenced by *Lc*S administration. Baseline data for zonulin and calprotectin are shown in Table 3.

**Table 3:** Zonulin and Calprotectin concentrations in serum and stool of MetS patients and controls. Data are given as median (quartiles).

	MetS patients (n = 28)	Normal range#
Serum zonulin (ng/ml)	45 (39–50)	52 (47–60)
Serum calprotectin (ng/ml)	555 (432.5–771.3)	570 (490–1050)
Stool zonulin (ng/ml)	75 (55–105)	31 (26–38) ***
Stool calprotectin (μg/ml)	29 (14.1–102.5)	25*

# Data from 11 healthy controls for serum parameters, data from 25 healthy controls for zonulin in stool, median of healthy controls from the kit description for calprotectin

*** p<0.001 compared to MetS

* p = 0.017 compared to MetS.

### Bile acids

Total bile acids in serum were within the normal range in 26 out of 28 patients. In two patients, total bile acids were slightly elevated (8.2 and 6.9μmol/l; upper limit of normal: 6.5μmol/). No difference in individual bile acids or total bile acids was found between patients with MetS and healthy controls. Proportions of primary (cholic acid, CA; chenodeoxycholic acid, CDCA) and secondary (deoxycholic acid, DCA; litocholic acid, LCA; ursodeoxycholic acid, UDCA) as well as proportions of taurine- or glycine conjugated bile acids were unaltered in MetS when compared to healthy controls. Absolute and relative amounts of individual bile acids, primary, secondary or proportions of taurine- or glycine conjugated bile acids did not change after consumption of *Lc*S for 12 weeks. No compelling evidence for a correlation between serum bile acids and markers of gut barrier disruption and inflammation was found.

## Discussion

We found that the *Bacteroidetes/Firmicutes* ratio, markers of gut barrier disruption and inflammation of patients with MetS differ significantly from healthy controls. Supplementation with *Lc*S increased the abundance of *Parabacteroides* but did not influence any other markers investigated in this study. Serum bile acid profile in MetS was not different to healthy controls.

We could confirm previously published findings of increased *Firmicutes* and decreased *Bacteroidetes* in obesity and MetS. [35–38] The gut microbiota has been shown to influence energy extraction from diet, regulation of lipogenesis and innate immunity and therefore plays an important role in the pathogenesis of MetS. [39] We could find the presence of two genera (*Bacteroides* and *Prevotella*) remaining stable over the study period and not influenced by *Lc*S. This confirms previous findings, showing no changes of the two genera, that other authors defined as enterotypes, during a 6-months controlled diet intervention. [9, 40]

An interesting species–*Akkermansia muciniphila*–has been associated with weight gain, insulin resistance and inflammatory changes. [41, 42] In our study we were not able to reproduce this finding.

The supplementation with *Lc*S led to a significant increase in *Parabacteroides* (phylum *Bacteroidetes)*. From our data we cannot explain this finding, we can only hypothesize that *Lc*S might lead to ecological rearrangements in gut microbiota leading to this increase in *Parabacteroides*. *Parabacteroides* derive energy mainly from fermentation from carbohydrates or proteins, however the amount of carbohydrates in the milk drink is probably too small to be responsible for this increase. It is unclear, if an increase of *Parabacteroides* is beneficial. A shift from *Firmicutes* towards *Bacteroidetes* could be seen as beneficial in metabolic disorders [43] and dietary supplementation of resistant starch has been shown to increase *Parabacteroides* [44], whereas a reduction was associated with recurrence in Crohn´s disease. [45] Contrary, in non-alcoholic steatohepatitis, a higher abundance of *Parabacteroides* was observed. [46] Since we previously published that supplementation with *Lc*S did not influence clinical and biochemical parameters of glucose metabolism, inflammation and innate immune response [20, 21], the relevance of our observation is unclear and cannot be answered by our study design. For more detailed information concerning abundance of gut microbiota see S2 Table.

We have chosen to study *Lactobacillus casei* Shirota for several reasons. This commercially available milk drink delivers a relatively high bacterial number in a relatively small volume. *Lc*S has been proven to survive the passage through the stomach and is still present in the lower intestinal tract [47–49]. Within previous studies *Lc*S appeared to be effective in modulating natural killer cell [50] and neutrophil function [51]. Previous studies detected *Lc*S in stool (both by PCR and culture) already after 7 days of ingestion of an *Lc*S containing milk drink [52–54]. In those studies, different milk drinks with 5–15 times higher daily concentrations of *Lc*S compared to our study were used. *Lc*S was also detected in stool of European subjects, taking the same product we used in our study, using culture techniques for *Lc*S detection. [55, 56] With the methodology we used we could not detect an increase in *Lactobacillus* genera by probiotic supplementation in stool samples. This is not unexpected considering the number of reads, the concentration of the product (6.5 x 10^9^ per day) and the dilution of the probiotic milk drink in the gut with a bacterial count of about 10^14^ cells. Therefore methodological limitations (limited number of reads per sample), differences in patient population and/or in the product can explain the different findings between our study and previous data.

In animal models increased gut permeability is associated with increased translocation of bacterial products and contributes to insulin resistance. [17] In a previous study we could show that subjects with MetS have increased gastroduodenal and small intestinal permeability compared to healthy controls. [20] Zonulin is the only physiological mediator known to regulate intestinal permeability reversibly by modulating intercellular tight junctions [57–59] and a positive correlation between zonulin, obesity and insulin resistance has been found recently. [60] Calprotectin, a protein expressed in neutrophil granulocytes, is a marker of intestinal inflammation. In accordance with our previous finding of increased gut permeability in MetS [20], zonulin and calprotectin levels were significantly elevated in stool but similar in serum of MetS patients compared to healthy controls. In contrast to animal and human studies that showed decreased faecal calprotectin levels by administration of a probiotic [61, 62], we could not find any influence of *Lc*S supplementation on zonulin or calprotectin levels.

Bile acids have been proposed as potential modulators of gut permeability. In a mouse model high fat diet has been shown to decrease UDCA but increase DCA. In this model DCA increased gut permeability by a direct, non-inflammatory mechanism. [13, 14] *Ex vivo* data suggest that selected bile acids modulate intestinal permeability via rearrangement at the tight junction level.[63] In contrast to high fat diet models, genetically obese mice did not show increased gut permeability or bile acid pool hydrophobicity. [64] Furthermore, bile acids may directly impact on glucose metabolism. Dietary increase of bile acid pool size in a rat model resulted in a reduction of fat mass through an increase in energy expenditure. [65] A reduction of bile acid pool size increased body weight gain and worsened glucose intolerance induced by the high fat diet and led to a pronounced worsening of the changes in liver and adipose tissue. [66]

Human data are scarce. A biosynthetic bile acid precursor is increased in patients with MetS and T2DM, but this study did not investigate gut permeability. [67]. We could not find differences in the serum bile acid profiles between MetS patients and healthy controls. Bile acid composition was not influenced by *Lc*S treatment. Unfortunately we were not able to analyze bile acids in stool due to lack of material.

Our study has some limitations: Due to the small sample size individual differences might have outweighed the effects of *Lc*S administration. Furthermore our healthy controls were significantly younger than our patient cohort. Age impacts on gut microbiota composition, but during adulthood the microbiota composition is relatively stable. [68] The *Bacteroidetes/Firmicutes* ratio decreases after infancy and rises again over the age of 70, but is reported to be stable in the age range of our controls and patients. [69]

This study suggests that investigating gut microbiota composition is challenging and interventions are difficult. Further studies are necessary to unravel the underlying mechanisms and find targeted therapeutic approaches for the complex interplay of gut microbiota and host metabolism.

## Supporting Information

S1 CONSORT Checklist (DOCX)

S1 Study Protocol (PDF)

S1 Table Barcoded primer sequences used in this study. Amplicons were sequenced from the Titanium A adaptor (CCATCTCATCCCTGCGTGTCTCCGAC), followed by a 4 bases key sequence (TCAG) and the 10 bases barcode. The reverse primer was used with the Titanium B adaptor (CCTATCCCCTGTGTGCCTTGGCAGTC), the key sequence and the target specific sequence but without barcode sequence (CCTATCCCCTGTGTGCCTTGGCAGTC TCAG ATTACCGCGGCTGCTGG).
(DOCX)

S2 Table Abundance of gut microbiota concerning *Lc*S supplementation. Median abundance is given for each group.
(DOCX)

## References

[1] WHO. Obesity and overweight. Fact sheet. 2014;131.

[2] Thomas GN, Schooling CM, McGhee SM, Ho SY, Cheung BM, Wat NM, et al Metabolic syndrome increases all-cause and vascular mortality: the Hong Kong Cardiovascular Risk Factor Study. Clinical endocrinology. 2007;66(5):666–71. 10.1111/j.1365-2265.2007.02798.x . 17381490

[3] Prospective Studies Collaboration, Whitlock G, Lewington S, Sherliker P, Clarke R, Emberson J, et al Body-mass index and cause-specific mortality in 900 000 adults: collaborative analyses of 57 prospective studies. Lancet. 2009;373(9669):1083–96. 10.1016/S0140-6736(09)60318-4 19299006 PMC2662372

[4] Esteve E, Ricart W, Fernandez-Real JM. Gut microbiota interactions with obesity, insulin resistance and type 2 diabetes: did gut microbiote co-evolve with insulin resistance? Current opinion in clinical nutrition and metabolic care. 2011;14(5):483–90. 10.1097/MCO.0b013e328348c06d . 21681087

[5] Ley RE, Backhed F, Turnbaugh P, Lozupone CA, Knight RD, Gordon JI. Obesity alters gut microbial ecology. Proc Natl Acad Sci U S A. 2005;102(31):11070–5. . 16033867 10.1073/pnas.0504978102 PMC1176910

[6] Backhed F, Ley RE, Sonnenburg JL, Peterson DA, Gordon JI. Host-bacterial mutualism in the human intestine. Science. 2005;307(5717):1915–20. . 15790844 10.1126/science.1104816

[7] Duncan SH, Lobley GE, Holtrop G, Ince J, Johnstone AM, Louis P, et al Human colonic microbiota associated with diet, obesity and weight loss. Int J Obes (Lond). 2008;32(11):1720–4. Epub 2008/09/10. 10.1038/ijo.2008.155 ijo2008155 [pii]. . 18779823

[8] Schwiertz A, Taras D, Schafer K, Beijer S, Bos NA, Donus C, et al Microbiota and SCFA in lean and overweight healthy subjects. Obesity (Silver Spring). 2010;18(1):190–5. Epub 2009/06/06. 10.1038/oby.2009.167 oby2009167 [pii]. . 19498350

[9] Zupancic ML, Cantarel BL, Liu Z, Drabek EF, Ryan KA, Cirimotich S, et al Analysis of the gut microbiota in the old order Amish and its relation to the metabolic syndrome. PLoS One. 2012;7(8):e43052 Epub 2012/08/21. 10.1371/journal.pone.0043052 PONE-D-11-23689 [pii]. 22905200 PMC3419686

[10] Zhang H, DiBaise JK, Zuccolo A, Kudrna D, Braidotti M, Yu Y, et al Human gut microbiota in obesity and after gastric bypass. Proc Natl Acad Sci U S A. 2009;106(7):2365–70. Epub 2009/01/24. 10.1073/pnas.0812600106 0812600106 [pii]. 19164560 PMC2629490

[11] Creely SJ, McTernan PG, Kusminski CM, Fisher M, Da Silva NF, Khanolkar M, et al Lipopolysaccharide activates an innate immune system response in human adipose tissue in obesity and type 2 diabetes. American journal of physiologyEndocrinology and metabolism. 2007;292(3):E740–7. 10.1152/ajpendo.00302.2006 17090751

[12] Larsen N, Vogensen FK, van den Berg FW, Nielsen DS, Andreasen AS, Pedersen BK, et al Gut microbiota in human adults with type 2 diabetes differs from non-diabetic adults. PLoS One. 2010;5(2):e9085 10.1371/journal.pone.0009085 20140211 PMC2816710

[13] Stenman LK, Holma R, Eggert A, Korpela R. A novel mechanism for gut barrier dysfunction by dietary fat: epithelial disruption by hydrophobic bile acids. Am J Physiol Gastrointest Liver Physiol. 2013;304(3):G227–34. Epub 2012/12/04. 10.1152/ajpgi.00267.2012 ajpgi.00267.2012 [pii]. . 23203158

[14] Stenman LK, Holma R, Korpela R. High-fat-induced intestinal permeability dysfunction associated with altered fecal bile acids. World J Gastroenterol. 2012;18(9):923–9. Epub 2012/03/13. 10.3748/wjg.v18.i9.923 22408351 PMC3297051

[15] Cani PD, Delzenne NM. The role of the gut microbiota in energy metabolism and metabolic disease. Current pharmaceutical design. 2009;15(13):1546–58. . 19442172 10.2174/138161209788168164

[16] Cani PD, Delzenne NM. Interplay between obesity and associated metabolic disorders: new insights into the gut microbiota. Current opinion in pharmacology. 2009;9(6):737–43. 10.1016/j.coph.2009.06.016 . 19628432

[17] Cani PD, Bibiloni R, Knauf C, Waget A, Neyrinck AM, Delzenne NM, et al Changes in gut microbiota control metabolic endotoxemia-induced inflammation in high-fat diet-induced obesity and diabetes in mice. Diabetes. 2008;57(6):1470–81. 10.2337/db07-1403 18305141

[18] Naito E, Yoshida Y, Makino K, Kounoshi Y, Kunihiro S, Takahashi R, et al Beneficial effect of oral administration of Lactobacillus casei strain Shirota on insulin resistance in diet-induced obesity mice. J Appl Microbiol. 2011;110(3):650–7. 10.1111/j.1365-2672.2010.04922.x 21281408

[19] Andreasen AS, Larsen N, Pedersen-Skovsgaard T, Berg RM, Moller K, Svendsen KD, et al Effects of Lactobacillus acidophilus NCFM on insulin sensitivity and the systemic inflammatory response in human subjects. Br J Nutr. 2010;104(12):1831–8. 10.1017/S0007114510002874 . 20815975

[20] Leber B, Tripolt NJ, Blattl D, Eder M, Wascher TC, Pieber TR, et al The influence of probiotic supplementation on gut permeability in patients with metabolic syndrome: an open label, randomized pilot study. Eur J Clin Nutr. 2012;66(10):1110–5. 10.1038/ejcn.2012.103 . 22872030

[21] Tripolt NJ, Leber B, Blattl D, Eder M, Wonisch W, Scharnagl H, et al Short communication: Effect of supplementation with Lactobacillus casei Shirota on insulin sensitivity, beta-cell function, and markers of endothelial function and inflammation in subjects with metabolic syndrome-A pilot study. J Dairy Sci. 2012 Epub 2012/11/21. S0022-0302(12)00847-8 [pii] 10.3168/jds.2012-5863 . 23164226

[22] Alberti KG, Eckel RH, Grundy SM, Zimmet PZ, Cleeman JI, Donato KA, et al Harmonizing the metabolic syndrome: a joint interim statement of the International Diabetes Federation Task Force on Epidemiology and Prevention; National Heart, Lung, and Blood Institute; American Heart Association; World Heart Federation; International Atherosclerosis Society; and International Association for the Study of Obesity. Circulation. 2009;120(16):1640–5. 10.1161/circulationaha.109.192644 19805654

[23] Baker GC, Smith JJ, Cowan DA. Review and re-analysis of domain-specific 16S primers. J Microbiol Methods. 2003;55(3):541–55. . 14607398 10.1016/j.mimet.2003.08.009

[24] Watanabe K, Kodama Y, Harayama S. Design and evaluation of PCR primers to amplify bacterial 16S ribosomal DNA fragments used for community fingerprinting. J Microbiol Methods. 2001;44(3):253–62. . 11240048 10.1016/s0167-7012(01)00220-2

[25] Kump PK, Grochenig HP, Lackner S, Trajanoski S, Reicht G, Hoffmann KM, et al Alteration of intestinal dysbiosis by fecal microbiota transplantation does not induce remission in patients with chronic active ulcerative colitis. Inflammatory bowel diseases. 2013;19(10):2155–65. 10.1097/MIB.0b013e31829ea325 . 23899544

[26] Stojakovic T, Putz-Bankuti C, Fauler G, Scharnagl H, Wagner M, Stadlbauer V, et al Atorvastatin in patients with primary biliary cirrhosis and incomplete biochemical response to ursodeoxycholic acid. Hepatology. 2007;46(3):776–84. Epub 2007/08/03. 10.1002/hep.21741 . 17668874

[27] Huse SM, Welch DM, Morrison HG, Sogin ML. Ironing out the wrinkles in the rare biosphere through improved OTU clustering. Environ Microbiol. 2010;12(7):1889–98. 10.1111/j.1462-2920.2010.02193.x 20236171 PMC2909393

[28] Caporaso JG, Kuczynski J, Stombaugh J, Bittinger K, Bushman FD, Costello EK, et al QIIME allows analysis of high-throughput community sequencing data. Nature methods. 2010;7(5):335–6. 10.1038/nmeth.f.303 20383131 PMC3156573

[29] Edgar RC. Search and clustering orders of magnitude faster than BLAST. Bioinformatics. 2010;26(19):2460–1. 10.1093/bioinformatics/btq461 . 20709691

[30] Wang Q, Garrity GM, Tiedje JM, Cole JR. Naive Bayesian classifier for rapid assignment of rRNA sequences into the new bacterial taxonomy. Appl Environ Microbiol. 2007;73(16):5261–7. 10.1128/AEM.00062-07 17586664 PMC1950982

[31] McDonald D, Price MN, Goodrich J, Nawrocki EP, DeSantis TZ, Probst A, et al An improved Greengenes taxonomy with explicit ranks for ecological and evolutionary analyses of bacteria and archaea. ISME J. 2012;6(3):610–8. 10.1038/ismej.2011.139 22134646 PMC3280142

[32] Caporaso JG, Bittinger K, Bushman FD, DeSantis TZ, Andersen GL, Knight R. PyNAST: a flexible tool for aligning sequences to a template alignment. Bioinformatics. 2010;26(2):266–7. 10.1093/bioinformatics/btp636 19914921 PMC2804299

[33] Haas BJ, Gevers D, Earl AM, Feldgarden M, Ward DV, Giannoukos G, et al Chimeric 16S rRNA sequence formation and detection in Sanger and 454-pyrosequenced PCR amplicons. Genome research. 2011;21(3):494–504. 10.1101/gr.112730.110 21212162 PMC3044863

[34] Price MN, Dehal PS, Arkin AP. FastTree 2—approximately maximum-likelihood trees for large alignments. PLoS One. 2010;5(3):e9490 10.1371/journal.pone.0009490 20224823 PMC2835736

[35] Backhed F, Manchester JK, Semenkovich CF, Gordon JI. Mechanisms underlying the resistance to diet-induced obesity in germ-free mice. Proc Natl Acad Sci U S A. 2007;104(3):979–84. 10.1073/pnas.0605374104 17210919 PMC1764762

[36] Ley RE. Obesity and the human microbiome. Current opinion in gastroenterology. 2010;26(1):5–11. 10.1097/MOG.0b013e328333d751 19901833

[37] Tilg H, Kaser A. Gut microbiome, obesity, and metabolic dysfunction. The Journal of clinical investigation. 2011;121(6):2126–32. doi: 10.1172/jci58109; 10.1172/jci58109 21633181 10.1172/JCI58109 PMC3104783

[38] Tremaroli V, Backhed F. Functional interactions between the gut microbiota and host metabolism. Nature. 2012;489(7415):242–9. 10.1038/nature11552 . 22972297

[39] Parekh PJ, Arusi E, Vinik AI, Johnson DA. The Role and Influence of Gut Microbiota in Pathogenesis and Management of Obesity and Metabolic Syndrome. Front Endocrinol (Lausanne). 2014;5:47 Epub 2014/04/30. 10.3389/fendo.2014.00047 . 24778627 PMC3984999

[40] Roager HM, Licht TR, Poulsen SK, Larsen TM, Bahl MI. Microbial enterotypes, inferred by the prevotella-to-bacteroides ratio, remained stable during a 6-month randomized controlled diet intervention with the new nordic diet. Appl Environ Microbiol. 2014;80(3):1142–9. 10.1128/AEM.03549-13 24296500 PMC3911217

[41] Qin J, Li Y, Cai Z, Li S, Zhu J, Zhang F, et al A metagenome-wide association study of gut microbiota in type 2 diabetes. Nature. 2012;490(7418):55–60. 10.1038/nature11450 . 23023125

[42] Fukuda S, Ohno H. Gut microbiome and metabolic diseases. Seminars in immunopathology. 2014;36(1):103–14. 10.1007/s00281-013-0399-z . 24196453

[43] Ley RE, Backhed F, Turnbaugh P, Lozupone CA, Knight RD, Gordon JI. Obesity alters gut microbial ecology. Proc Natl Acad Sci U S A. 2005;102(31):11070–5. 10.1073/pnas.0504978102 16033867 PMC1176910

[44] Martinez I, Kim J, Duffy PR, Schlegel VL, Walter J. Resistant starches types 2 and 4 have differential effects on the composition of the fecal microbiota in human subjects. PLoS One. 2010;5(11):e15046 10.1371/journal.pone.0015046 21151493 PMC2993935

[45] De Cruz P, Kang S, Wagner J, Buckley M, Sim WH, Prideaux L, et al Specific Mucosa-Associated Microbiota in Crohn's Disease at the Time of Resection are Associated with Early Disease Recurrence: A Pilot Study. Journal of gastroenterology and hepatology. 2014 10.1111/jgh.12694 25087692

[46] Wong VW, Tse CH, Lam TT, Wong GL, Chim AM, Chu WC, et al Molecular characterization of the fecal microbiota in patients with nonalcoholic steatohepatitis—a longitudinal study. PLoS One. 2013;8(4):e62885 10.1371/journal.pone.0062885 23638162 PMC3636208

[47] Shirota M, Aso K, Iwabuchi A. Studies on intestinal microflora. 1. Its constitution in healthy infants and the effect of oral administration of L. acidophilus strain Shirota. Nippon saikingaku zasshiJapanese journal of bacteriology. 1966;21(5):274–83. 4959326

[48] Spanhaak S, Havenaar R, Schaafsma G. The effect of consumption of milk fermented by Lactobacillus casei strain Shirota on the intestinal microflora and immune parameters in humans. Eur J Clin Nutr. 1998;52(12):899–907. 9881885 10.1038/sj.ejcn.1600663

[49] Tuohy KM, Pinart-Gilberga M, Jones M, Hoyles L, McCartney AL, Gibson GR. Survivability of a probiotic Lactobacillus casei in the gastrointestinal tract of healthy human volunteers and its impact on the faecal microflora. J Appl Microbiol. 2007;102(4):1026–32. 10.1111/j.1365-2672.2006.03154.x 17381746

[50] Takeda K, Okumura K. Effects of a fermented milk drink containing Lactobacillus casei strain Shirota on the human NK-cell activity. The Journal of nutrition. 2007;137(3 Suppl 2):791S–3S. 17311976 10.1093/jn/137.3.791S

[51] Stadlbauer V, Mookerjee RP, Hodges S, Wright GA, Davies NA, Jalan R. Effect of probiotic treatment on deranged neutrophil function and cytokine responses in patients with compensated alcoholic cirrhosis. J Hepatol. 2008;48(6):945–51. 10.1016/j.jhep.2008.02.015 18433921

[52] Tiengrim S, Leelaporn A, Manatsathit S, Thamlikitkul V. Viability of Lactobacillus casei strain Shirota (LcS) from feces of Thai healthy subjects regularly taking milk product containing LcS. Journal of the Medical Association of Thailand = Chotmaihet thangphaet. 2012;95 Suppl 2:S42–7. Epub 2012/05/12. . 22574528

[53] Yuki N, Watanabe K, Mike A, Tagami Y, Tanaka R, Ohwaki M, et al Survival of a probiotic, Lactobacillus casei strain Shirota, in the gastrointestinal tract: selective isolation from faeces and identification using monoclonal antibodies. International journal of food microbiology. 1999;48(1):51–7. Epub 1999/06/22. . 10375134 10.1016/s0168-1605(99)00029-x

[54] Fujimoto J, Matsuki T, Sasamoto M, Tomii Y, Watanabe K. Identification and quantification of Lactobacillus casei strain Shirota in human feces with strain-specific primers derived from randomly amplified polymorphic DNA. International journal of food microbiology. 2008;126(1–2):210–5. 10.1016/j.ijfoodmicro.2008.05.022 . 18573558

[55] Sakai T, Oishi K, Asahara T, Takada T, Yuki N, Matsumoto K, et al M-RTLV agar, a novel selective medium to distinguish Lactobacillus casei and Lactobacillus paracasei from Lactobacillus rhamnosus. International journal of food microbiology. 2010;139(3):154–60. 10.1016/j.ijfoodmicro.2010.03.019 . 20385416

[56] Tilley L, Keppens K, Kushiro A, Takada T, Sakai T, Vaneechoutte M, et al A probiotic fermented milk drink containing Lactobacillus cassei strain Shirota improves stool consistency of subjects with hard stools. International Journal of Probiotics and Prebiotics. 2014;9(1–2):23–30.

[57] Fasano A. Regulation of intercellular tight junctions by zonula occludens toxin and its eukaryotic analogue zonulin. Ann N Y Acad Sci. 2000;915:214–22. Epub 2001/02/24. . 11193578 10.1111/j.1749-6632.2000.tb05244.x

[58] Wang W, Uzzau S, Goldblum SE, Fasano A. Human zonulin, a potential modulator of intestinal tight junctions. J Cell Sci. 2000;113 Pt 24:4435–40. Epub 2000/11/18. . 11082037 10.1242/jcs.113.24.4435

[59] Fasano A, Not T, Wang W, Uzzau S, Berti I, Tommasini A, et al Zonulin, a newly discovered modulator of intestinal permeability, and its expression in coeliac disease. Lancet. 2000;355(9214):1518–9. Epub 2000/05/09. S0140-6736(00)02169-3 [pii] 10.1016/S0140-6736(00)02169-3 . 10801176

[60] Moreno-Navarrete JM, Sabater M, Ortega F, Ricart W, Fernandez-Real JM. Circulating zonulin, a marker of intestinal permeability, is increased in association with obesity-associated insulin resistance. PLoS One. 2012;7(5):e37160 Epub 2012/05/26. 10.1371/journal.pone.0037160 PONE-D-12-01867 [pii]. 22629362 PMC3356365

[61] Goodrich KM, Fundaro G, Griffin LE, Grant A, Hulver MW, Ponder MA, et al Chronic administration of dietary grape seed extract increases colonic expression of gut tight junction protein occludin and reduces fecal calprotectin: a secondary analysis of healthy Wistar Furth rats. Nutr Res. 2012;32(10):787–94. Epub 2012/11/14. 10.1016/j.nutres.2012.09.004 S0271-5317(12)00186-8 [pii]. . 23146776

[62] Vulevic J, Juric A, Tzortzis G, Gibson GR. A mixture of trans-galactooligosaccharides reduces markers of metabolic syndrome and modulates the fecal microbiota and immune function of overweight adults. J Nutr. 2013;143(3):324–31. Epub 2013/01/11. 10.3945/jn.112.166132 jn.112.166132 [pii]. . 23303873

[63] Raimondi F, Santoro P, Barone MV, Pappacoda S, Barretta ML, Nanayakkara M, et al Bile acids modulate tight junction structure and barrier function of Caco-2 monolayers via EGFR activation. Am J Physiol Gastrointest Liver Physiol. 2008;294(4):G906–13. Epub 2008/02/02. 10.1152/ajpgi.00043.2007 . 18239063

[64] Stenman LK, Holma R, Gylling H, Korpela R. Genetically obese mice do not show increased gut permeability or faecal bile acid hydrophobicity. Br J Nutr. 2013;110(6):1157–64. Epub 2013/02/28. 10.1017/S000711451300024X . 23442231

[65] Liaset B, Hao Q, Jorgensen H, Hallenborg P, Du ZY, Ma T, et al Nutritional regulation of bile acid metabolism is associated with improved pathological characteristics of the metabolic syndrome. The Journal of biological chemistry. 2011;286(32):28382–95. Epub 2011/06/18. 10.1074/jbc.M111.234732 21680746 PMC3151081

[66] Watanabe M, Horai Y, Houten SM, Morimoto K, Sugizaki T, Arita E, et al Lowering bile acid pool size with a synthetic farnesoid X receptor (FXR) agonist induces obesity and diabetes through reduced energy expenditure. The Journal of biological chemistry. 2011;286(30):26913–20. Epub 2011/06/03. 10.1074/jbc.M111.248203 21632533 PMC3143650

[67] Steiner C, Othman A, Saely CH, Rein P, Drexel H, von Eckardstein A, et al Bile acid metabolites in serum: intraindividual variation and associations with coronary heart disease, metabolic syndrome and diabetes mellitus. PLoS One. 2011;6(11):e25006 Epub 2011/11/24. 10.1371/journal.pone.0025006 22110577 PMC3215718

[68] Voreades N, Kozil A, Weir TL. Diet and the development of the human intestinal microbiome. Frontiers in microbiology. 2014;5:494 10.3389/fmicb.2014.00494 25295033 PMC4170138

[69] Mariat D, Firmesse O, Levenez F, Guimaraes V, Sokol H, Dore J, et al The Firmicutes/Bacteroidetes ratio of the human microbiota changes with age. BMC Microbiol. 2009;9:123 10.1186/1471-2180-9-123 19508720 PMC2702274

