# Supplementary material for: Lactobacillus casei Shirota Supplementation Does Not Restore Gut Microbiota Composition and Gut Barrier in Metabolic Syndrome: A Randomized Pilot Study
Source: PLoS One. 2015 Oct 28;10(10):e0141399. doi: 10.1371/journal.pone.0141399 (PMC4625062; doi:10.1371/journal.pone.0141399)
Supplement: S1 Study Protocol — (PDF) [file pone.0141399.s002.pdf]

Study Protocol

Version 5

Obesity – Inflammation - Metabolic Disease: Effect of Lactobacillus casei Shirota

Date: 18.3.2014

Principal Investigators

Assoz. Prof. Dr. Vanessa Stadlbauer-Köllner
Division of Gastroenterology and Hepatology, Department of Internal Medicine, Medical University of Graz
Assoz.Prof. Dr. Harald Sourij
Division of Endocrinology and Nuclear Medicine, Department of Internal Medicine, Medical University of Graz

Co-Investigators

Prof. Dr. Christoph Högenauer
Division of Gastroenterology and Hepatology, Department of Internal Medicine, Medical University of Graz
Prof. Dr. Thomas Wascher
Department of Internal Medicine, Hanusch Hospital Vienna

SYNOPSIS

DATE OF PROTOCOL	13.8.2014
SHORT TITLE	LcS for metabolic syndrome
VERSION	5
BACKGROUND	Obesity and metabolic syndrome are linked by inflammation. Gut flora seems to play an important role in the development of inflammation and metabolic syndrome in obesity. Modulation of gut flora by probiotics has been shown in animal studies to positively influence inflammation and metabolic disturbances.
HYPOTHESIS	Lactobacillus casei Shirota is able to decrease metabolic endotoxemia by altering gut flora composition and gut permeability which leads to an improvement in neutrophil function and insulin resistance in obesity
SPECIFIC AIMS	1) To investigate the effect of Lactobacillus casei Shirota supplementation over 12 weeks on neutrophil function (phagocytosis, oxidative burst and TLR expression) in patients with metabolic syndrome.<br>2) To investigate the effect of Lactobacillus casei Shirota supplementation over 12 weeks on glucose tolerance, insulin resistance, inflammation, gut flora composition, gut permeability, and endotoxemia in metabolic syndrome
NUMBER OF PATIENTS	30
PATIENT SELECTION	Consecutive patients with metabolic syndrome
DURATION OF STUDY	Start of study 01/2009<br>End of study 11/2010

INDEX

Introduction 4
- Gut flora interactions 4
- Role of endotoxin 4
- Consequences of chronic inflammation in obesity 5
- Effects of modulating gut flora 5
- Which probiotic? 5

Hypothesis 6
Specific Aims 6
Plan of investigations 6
- Patients 6
- Inclusion and exclusion criteria 7
- Randomization 8
- *Study flow and management of patients* 8
- End of the study 8
- Endpoints 8

Study flowchart 10
Methods 11
References 16

Introduction

Obesity and metabolic disorders (type 2 diabetes and insulin resistance) are tightly linked to inflammation (1) Obesity, a pandemic affecting 30-50% of the adult population, is mediated by a variety of genetic and environmental factors. (2) It is well described that cytokines cause insulin resistance which causes hyperinsulinemia and excessive fat storage in adipose tissue and the liver. (3) However, the triggering factor, linking inflammation to metabolic syndrome has not been fully elucidated yet.

Gut flora interactions

Recently it has been hypothesized that the gut flora is an important factor in this vicious cycle of obesity, metabolic disease and inflammation. Firstly, metabolic activities of the gut microbiota facilitates the extraction of calories from ingested dietary substances and helps to store these calories in host adipose tissue for later use. (4-6) Second, the gut bacterial flora of obese mice and humans include fewer Bacteroidetes and correspondingly more Firmicutes than that of their lean counterparts, suggesting that differences in caloric extraction of ingested food substances may be due to the composition of the gut microbiota. (7-9) Furthermore, bacterial lipopolysaccharide derived from the intestinal microbiota may trigger inflammation, linking it to high-fat diet-induced metabolic syndrome. High-fat diet induces insulin resistance and oxidative stress in mice and is associated with increased gut permeability. (10) high fat diet induces a low-grade endotoxemia in mice ("metabolic endotoxemia) and infusing endotoxin causes weight gain and insulin resistance. (11) This has also been shown in humans, where patients with fatty liver had a susceptibility to higher gut permeability, possibly causing increased endotoxin levels. (12)

Role of endotoxin

Endotoxin and Lipopolysaccharide-binding protein (LBP) is elevated in obese patients, patients with type 2 diabetes and patients with liver steatosis. (13, 14) Endotoxin causes a significant increase in proinflammatory cytokine production in adipocytes via a TLR mediated pathway, contribution to the proinflammatory state in obesity. (13, 14) Endotoxin levels correlate with adiponectin (15) and insulin (13, 14) suggesting a pathophysiological link between obesity, inflammation and metabolic disease.

Consequences of chronic inflammation in obesity

As described above, endotoxin is related to increased inflammation and oxidative stress, causing insulin resistance. Adipocytes have been shown to play a dynamic role in regulation of inflammation by producing cytokines via a Toll-like receptor (TLR)/Nuclear Factor kappa B (NFkB) mediated pathway. (16) But not only adipocytes are in a proinflammatory state – also circulating mononuclear cells have been described to be activated (17)

Clinical evidence suggests immune dysfunction in obesity, since obese patients are more prone to infections after surgery, higher incidence of lower respiratory infection which is also underlined by impairment of cell-mediated immune responses in vivo and in vitro and a reduced intracellular killing by neutrophils. (Reviewed in (18))

A similar situation has been recently described in alcoholic cirrhosis and alcoholic hepatitis, which is also a proinflammatory condition with impaired innate immunity, leading to infection. Endotoxin has been described as a key mediator and inadequate activation of neutrophils leading to high oxidative burst and energy depletion of the cells with consecutive impaired phagocytic capacity has been described. (19)

Effects of modulating gut flora

The most effective therapy of obesity – weight loss – leads to significant improvement of mononuclear cell activation. (20) However, there is no data available on the effect of weight loss on gut flora, gut permeability and endotoxin.

Since weight loss is usually very hard to achieve, other therapeutic strategies have been tested. Since gut flora seems to be crucial in the development of the vicious cycle of obesity, inflammation and metabolic disease, several studies tried to modify the composition of gut microbiota. In mice treatment with antibiotics improved glucose tolerance by altering expression of genes involved in inflammation and metabolism. (10, 21) A similar result was found in mice treated with a probiotic that increases the number of Bifidobacterium spp., which leads to improved glucose tolerance, insulin secretion and a decrease in inflammatory tone. (22) Finally treatment of mice with a probiotic (VSL#3) decreased hepatic insulin resistance via a JNK and NFkB pathway, supporting the concept that intestinal bacteria induce endogenous signals that play a pathogenic role in hepatic insulin resistance. (23)

Which probiotic?

Among the vast amount of bacteria described to alter gut flora and exert positive effects on the host, we have chosen to study Lactobacillus casei Shirota several reasons: Firstly this commercially available preparation delivers a high bacterial number in a relatively

small volume and is available as a palatable milk drink. Furthermore *Lactobacillus casei* Shirota has been proven to survive the passage through the stomach and is present in the lower intestinal tract (24-26). It has also been shown that this bacterial strain can increases the amount of Lactobacilli and decreases the number of gram-negative organisms in the bacterial flora (24, 27, 28). This bacterial strain has been shown to be effective in modulating natural killer cell function (29) and neutrophil function. (30)

Hypothesis

We hypothesize that Lactobacillus casei Shirota is able to decrease metabolic endotoxemia by altering gut flora composition and gut permeability which leads to an improvement in neutrophil function and insulin resistance in obesity

Specific Aims

1) To investigate intestinal permeability and its relation to systemic inflammation in patients with metabolic syndrome.
2) To investigate the effect of Lactobacillus casei Shirota supplementation over 12 weeks on neutrophil function (phagocytosis, oxidative burst and TLR expression) in patients with metabolic syndrome.
3) To investigate the effect of Lactobacillus casei Shirota supplementation over 12 weeks on glucose tolerance, insulin resistance, inflammation, gut flora composition, and endotoxemia in metabolic syndrome

Plan of investigations

Patients

30 Patients with metabolic syndrome will be randomized to either receive food supplementation with a milk drink containing Lactobacillus casei Shirota (3 bottles a day, 65 ml each, containing *Lactobacillus casei* Shirota at a concentration of 10^8/ml) for twelve weeks or standard medical therapy.

Inclusion and exclusion criteria

Inclusion criteria

- Age >18
- Informed consent
- Fasting blood glucose ≥100mg/dL
- Metabolic syndrome defined by the NCEP-ATPIII criteria (3 out of 5)
    - Abdominal obesity (waist circumference ≥102 in men or ≥88 in women)
    - Elevated blood pressure (≥135/≥85) or drug treatment for elevated blood pressure
    - Fasting blood glucose ≥100mg/dL or previously known type 2 diabetes mellitus,
    - HDL cholesterol <40 mg/dL (men) or <50 mg/dL (women) or drug treatment for low HDL cholesterol
    - Triglycerides ≥150 mg/dL or drug treatment for elevated for high triglyzerides
    -
- HbA1C ≤7.0%
- Increased gut permeability (lactulose/mannitol ratio >0.03)??

Exclusion Criteria:

- Drug treatment for diabetes mellitus
- Liver cirrhosis (biopsy proven) or elevated transaminases (≥2x ULN)
- Inflammatory bowel disease (Crohns disease, ulcerative colitis)
- Celiac disease
- Alcohol abuse (more than 40g alcohol per day in the history)
- clinical evidence of active infection
- antibiotic treatment within 7 days prior to enrolment
- use of immunomodulating agents within previous month (steroids etc.)
- concomitant use of supplements (pre-, pro-, or synbiotics) likely to influence the study
- Any severe illness unrelated to metabolic syndrome
- malignancy
- pregnancy

Randomization

The patients will be randomised using the "Randomizer" (IMI Graz) software.

Study flow and management of patients

Patients will be identified from the outpatient clinic at the Department of Medicine and enrolled into the study on an outpatient basis.
After fulfilling inclusion criteria, patients will be randomized to either receive food supplementation with Lactobacillus casei Shirota (3 bottles of Yakult™ light per day) or standard medical care.
Patients will be advised not to consume any other probiotic supplements during the study period. This will be ensured by handing out a checklist containing all commercially available probiotics in Austria to the patients.
At day 0 and at day 84 (12 weeks) patients will be seen in the outpatients clinic for a detailed examination. On day 28 and 56 the patients will be seen for a routine blood test and a physical examination.
Every two weeks the patients in the treatment group will visit the outpatients clinic to receive the milk drink.

Patients will be managed for their diabetes mellitus type 2 and the metabolic syndrome according to the current national guidelines (www.oedg.org). If patients have elevated liver function tests, a complete hepatological workup will be performed prior to the study to exclude any underlying liver disease other than steatosis.

End of the study

For the individual patient the study will end at day 84 or at the occurrence of a severe adverse event. The study will terminate after the last patient has finished the study. No interim analysis is planned

Endpoints

Neutrophil phagocytosis, neutrophil oxidative burst and TLR expression, glucose tolerance, insulin resistance, plasma and ex vivo stimulated cytokines, endotoxin, gut flora composition, gut permeability

After analysis of the first set of data, the remaining material will be used to assess gut permeability modulation and changes in gut microbiota in depth.

Study flowchart

Day	-21 to -1	0	28	56	84
**Visit no.**	**1**	**2**			**3**
Inclusion / exclusion criteria	X				
Demographics / relevant medical history / current medical conditions	X	X			X
Prior concomitant meds/therapies	X	X			X
Physical examination / BP, HR, resp rate	X	X			X
Body weight/ height	X	X			X
Food frequency questionnaire		X			X
Abdominal ultrasound and Fibroscan	X				X
Blood count, liver function tests, renal function tests, lipids, glucose, CRP and hsCRP, glucose, insulin	X	X	X	X	X
Oral glucose tolerance test		X			X
Urinary analysis for albumin and creatinine	X	X	X	X	X
Gut permeability test (Saccharose/Lactulose/Mannitol)	X	X			X
50 ml blood for neutrophil function, endotoxin, cytokines (lithium-heparin, EDTA, serum)		X			X
Stool sample		X			X
Dosage administration record	Ongoing data capture				
Adverse Events	Ongoing data capture				
Concomitant medications/therapies	Ongoing data capture				
Comments	Ongoing data capture				

Methods

Neutrophil phagocytosis

The Phagotest® (Orpegen Pharma, Heidelberg, Germany) is used to measure phagocytosis by using FITC-labelled opsonized *E. coli* bacteria as described before. (19)

Neutrophil oxidative burst

The Phagoburst® kit (Orpegen Pharma, Heidelberg, Germany) is used to determine the percentage of neutrophils that produce reactive oxidants with or without stimulation according to the manufacturer's instructions as previously described using fluorescence activated cell sorting (FACS; Becton Dickinson FACScan, San Jose, USA; Cellquest™ software) (19)

Neutrophil TLR expression:

Surface expression of TLRs are determined using the following fluorescein isothiocyanate (FITC)- and phycoerythrin (PE)-conjugated antibodies: TLR2-PE-Cy7, TLR4-APC, TLR9-PE, CD16-FITC (eBioscience, San Diego, CA). Cells are incubated with the respective antibody pairs for 30 minutes, washed twice with PBS, lysed and fixed before FACS analysis. For TLR9 expression cells are permeabilized by incubating for 10 minutes with 500 µL of 1 x FACS Permeabilization Solution 2 (BD Biosciences, UK). Results will be confirmed by western blotting

Gut permeability:

<u>Lactulose/Mannitol test</u>

The patient drinks a solution of 200 ml water containing 5g lactulose and 1g L-rhamnose. Urine is collected over 5 hours while fasting is continued for 3 hours after study start. The urine volume collected at 5 hours is measured and 1-ml aliquots are frozen immediately at -80°C without preservative for subsequent analysis by high performance liquid chromatography. The mobile phase is degassed acetonitrile in distilled deionized water (70/30 by vol) at a flow rate of 1 ml/min. Detection is performed by a refractive index detector (LC 1240 R.I. Detector; GBC Scientific Equipment, Dandenong, Australia).

<u>DAO ELISA</u>

A ready-to-use solid-phase sandwich ELISA (Immundiagnostik AG, Bensheim, Germany) is used to detect DAO in serum samples. The test is performed according to the manufacturer's instructions.

Briefly, standards, controls and samples are immobilized at the per-coated plate and then incubated with a detection antibody. After incubation with a conjugate, substrate solution is added. The reaction is stopped by addition of stop solution and measurement is done at 450nm versus 690nm as reference wavelength

Zonulin ELISA

A ready-to-use solid-phase sandwich ELISA (Immundiagnostik AG, Bensheim, Germany) is used to detect Calprotectin in serum samples. The test is performed according to the manufacturer's instructions. Briefly, standards, controls and samples are mixed with tracer and then immobilized at the per-coated plate. After incubated with conjugate substrate solution is added. The reaction is stopped by addition of stop solution and measurement is done at 450nm versus 690nm as reference wavelength.

Calprotectin ELISA

A ready-to-use solid-phase sandwich ELISA (Immundiagnostik AG, Bensheim, Germany) is used to detect Calprotectin in serum samples. The test is performed according to the manufacturer's instructions. Briefly, standards, controls and samples are immobilized at the per-coated plate and then incubated with a detection antibody. After incubation with a conjugate, substrate solution is added. The reaction is stopped by addition of stop solution and measurement is done at 450nm versus 690nm as reference wavelength.

Bile acid detection from stool or serum

All bile acids (cholic acid, deoxycholic acid, chenodeoxycholic acid, lithocholic acid, UDCA) were assessed as unconjugated acids and as taurine and glycine conjugates using a tandem mass spectrometry method as described previously (Stojakovic T., 2007, Hepatology). All subfractions of the bile acids (free acids and their corresponding conjugates) were analyzed by 3 different multiple-reaction monitoring experiments within 1 high-performance liquid chromatography run. High-performance liquid chromatography was performed on a reversed-phase (C18) column that used a methanol/water gradient for chromatographic solution of isobaric bile acids. Quantitation was done by the use of deuterated internal standards and correlation of peak area ratios in linear regression.

Glucose Metabolism

After an overnight fast a 3h-oral glucose tolerance test will be performed. Insulin, C-peptide and glucose will be measured before and 15, 30, 60, 120 and 180 minutes after ingestion of 75g glucose.

Insulin sensitivity will be assessed by homeostasis model assessment (HOMA, (FPG(mmol/l)xFSI(U/ml))/22.5) (31) as well as by the insulin sensitivity index (ISI; 2-0.00333xBMI-0.0000779xIns120-0.000422xage) as suggested by Stumvoll et al. (32) In addition the clamp like index (CLIX) will be calculated (serum creatinine (x0.85 if male)/(mean AUC(glucose) x mean AUC(C-peptide)) x 6,600) (33).
Beta-cell function is quantified as the ratio of the incremental insulin to glucose responses over the first 30 min during the OGTT on the one hand (DeltaI(30)/DeltaG(30)) (34) and by 1st phase and 2nd phase insulin secretion index on the other hand (1st phase=1283+1.829*Ins30-138.7*Gluc30+3.772*Ins0, 2nd phase=286+0.416*Ins30-25.94*Gluc30+0.926*Ins0). (32)

Gut microbiome and bacterial DNA in plasma

Isolation of bacterial DNA is done from stool samples or plasma samples using QIAGEN stool DNA extraction kit according to the manufacturer's instruction. 16S rDNA variable region 4 is amplified from these DNA isolates by PCR using a according primers. PCR is done in triplicates for each sample (á 20µl) according to the following protocol using Roche´s FAST Start High Fidelity PCR system. PCR products are separated on a 1% 1xTAE agarose gel and specific bands (~300bp) are excised and gel-extracted using QIAGEN gel extraction kit (QIAGEN, Vienna). Purified PCR products are analysed on a BioAnalyzer 2100 DNA 1000 cassetes (Agilent Technologies) for integrity and DNA concentration is determined fluorometrically using QuanitDect reagent (Invitrogen, Carlsbad, CA). An amplicon library is generated using aequimolar amounts of PCR products derived from the individual samples and bound to the sequencing beads at a one molecule per bead ratio. Long Read Sequencing using a 70x75 PicoTiter Plate (Roche Diagnostics) is done on a Genome Sequencer FLX system (Roche Diagnostics) according to the manufacturer's instruction to yield ~400,000 reads (~20,000 per sample) with an average reading length of 264 bases.
Data Analysis is done according to the following work flow: 1) Sort-out low quality sequences; pyrosequencing reads with N's. 2. Group sequences according to MIDs. 3. Multi-sequence alignment with NAST available as web-application on the Greengenes homepage (http://greengenes.lbl.gov/cgi-bin/nph-index.cgi). Make Excel file to define how many similar sequences have been found in each sample. 4. Place sequences into full length 16S neighbor-joining (NJ) tree with ARB. Existing ARB file can be found on Greengenes webpage. 5. Export full sequence, ARB generated tree (the pre-existing sequences have to be removed before) for statistical analysis of differences between samples (PCoA,..) using UniFrac. 6. (Remove alignment); sequences binned to 97% OTU's (collector-, rarefaction curves) by using DOTUR.

Plasma cytokines and ex vivo cytokine production

IL6 is analyzed by a chemoluminescent assay (Immulite 2000, DPC Biermann GmbH, Bad Nauheim, Germany). IL8, IL10, TNFα (Quantikine®, R&D Systems, Minneapolis, MN, USA) and sTNFαR1 and 2 (BMS203CE, Bender MedSystem, Vienna, Austria) are determined by ELISA. The methods were used according to the manufacturer's recommendations. In brief, for IL6 a solid-phase, enzyme-labeled, chemiluminescent sequential immunometric assay is performed. A 100-µl serum sample is added to the test tube containing an assay-specific coated bead and incubated at 37°C for 30 minutes. Unbound material is washed from the bead and chemiluminescent substrate was added. Light emission is read with a high-sensitivity photon counter. For cytokine ELISA (IL8, IL10, TNFα and sTNFαR1 and 2) analyte-specific antibodies (capture antibodies) are pre-coated onto a microplate. A 100-µl serum sample is added and any analyte present is bound by the immobilized antibody. An enzyme-linked analyte-specific detection antibody binds to a second epitope on the analyte, forming the analyte-antibody complex. Substrate is added and optical density is read on a microplate reader.
Ex vivo stimulation of cytokines will be analysed in a whole blood assay after stimulation with endotoxin derived from E.*coli* (*E.coli* 0111:B4 Lot 085K4068, Sigma, Poole, UK) for 4 h at 37 °C.

In addition hsCRP, sICAM-1, sVCAM-1, von Willebrand factor will be determined by ELISA as described above. According to the Hoorn-study publication (35), a summarizing z score for inflammation and endothelial dysfunction will be calculated: (individual value−the mean value for the study population)/standard deviation. The summarising score for inflammation is calculated as followed: (z score for CRP+z score for sICAM-1)/2.
Summarising score for endothelial dysfunction: z scores for vWf, sVCAM-1 and albumin creatinin ratio.

Measurements of choline metabolites

TMAO, trimethylamine, choline, betaine, and their d9-isotopologues are quantified with the use of a stable-isotope-dilution as-say and high-performance liquid chromatography with online electrospray ionization tandem mass spectrometry; d4(1,1,2,2)-choline, d3(methyl)-tri- methylamine-*N*-oxide, and d3(methyl)-trimethyl- amine are used as internal standards. Levels of TMAO in urine are adjusted for urinary dilution by analysis of the urine creatinine level.

Endotoxin (Limulus amoebocyte lysate assay) and related proteins

Endotoxin

Heparinized whole blood is drawn with pyrogen-free needles into pyrogen-free tubes and the serum separated at 4°C and stored at -80°C in pyrogen-free polyethylene cryotubes (Nunc, Rochester, USA) on the day of collection. The chromogenic limulus amoebocyte lysate assay (Charles River Laboratories) is used for detection of endotoxin. (36) To measure endotoxin-binding capacity, plasma samples are spiked with 0.005, 0.05, 0.5, 5 and 50 EU of endotoxin and the percentage of recovery at the different concentrations is measured. Plasma from healthy control subjects is used as a reference.

LBP ELISA

A ready-to-use solid-phase sandwich ELISA (Hycult biotechnology, Uden, Netherlands) is used to detect LBP levels in EDTA plasma samples. The test is performed according to the manufacturer's instructions. Briefly, samples and standards are bound to the pre-coated plate. Afterwards samples and standards are incubated with tracer antibody followed by a streptavidin-peroxidase solution. After incubation with TMB substrate the reaction is stopped by addition of stop solution and measurement is performed at 450nm.

sCD14 ELISA

A ready-to-use solid-phase sandwich ELISA (Hycult biotechnology, Uden, Netherlands) was used to detect sCD14 levels in EDTA plasma samples. The test is performed according to the manufacturer's instructions. Briefly, samples and standards are bound to the pre-coated plate. Afterwards samples and standards are incubated with tracer antibody followed by a streptavidin-peroxidase solution. After incubation with TMB substrate the reaction is stopped by addition of stop solution and measurement is performed at 450nm.

Statistical methodology and data analysis

Since this study is a pilot study, no formal sample size calculation can be performed.

All the data will be described as mean and standard errors. All clinical data will be analysed on an intention to treat basis but will also be described on 'as treated' basis. The primary analysis will be based on a t-test. Analysis of the secondary endpoints will be done as descriptive statistics, by t-test, Mann-Whitney test, Pearson and/or Spearman correlation as appropriate.

References

1. Wellen KE, Hotamisligil GS. Inflammation, stress, and diabetes. J Clin Invest 2005 May;115(5):1111-1119.
2. Yanovski SZ, Yanovski JA. Obesity. N Engl J Med 2002 Feb 21;346(8):591-602.
3. Tilg H, Moschen AR. Inflammatory mechanisms in the regulation of insulin resistance. Mol Med 2008 Mar-Apr;14(3-4):222-231.
4. Backhed F, Ding H, Wang T, Hooper LV, Koh GY, Nagy A, et al. The gut microbiota as an environmental factor that regulates fat storage. Proc Natl Acad Sci U S A 2004 Nov 2;101(44):15718-15723.
5. Backhed F, Manchester JK, Semenkovich CF, Gordon JI. Mechanisms underlying the resistance to diet-induced obesity in germ-free mice. Proc Natl Acad Sci U S A 2007 Jan 16;104(3):979-984.
6. Turnbaugh PJ, Ley RE, Mahowald MA, Magrini V, Mardis ER, Gordon JI. An obesity-associated gut microbiome with increased capacity for energy harvest. Nature 2006 Dec 21;444(7122):1027-1031.
7. Backhed F, Ley RE, Sonnenburg JL, Peterson DA, Gordon JI. Host-bacterial mutualism in the human intestine. Science 2005 Mar 25;307(5717):1915-1920.
8. Ley RE, Backhed F, Turnbaugh P, Lozupone CA, Knight RD, Gordon JI. Obesity alters gut microbial ecology. Proc Natl Acad Sci U S A 2005 Aug 2;102(31):11070-11075.
9. Ley RE, Turnbaugh PJ, Klein S, Gordon JI. Microbial ecology: human gut microbes associated with obesity. Nature 2006 Dec 21;444(7122):1022-1023.
10. Cani PD, Bibiloni R, Knauf C, Waget A, Neyrinck AM, Delzenne NM, et al. Changes in gut microbiota control metabolic endotoxemia-induced inflammation in high-fat diet-induced obesity and diabetes in mice. Diabetes 2008 Jun;57(6):1470-1481.
11. Cani PD, Amar J, Iglesias MA, Poggi M, Knauf C, Bastelica D, et al. Metabolic endotoxemia initiates obesity and insulin resistance. Diabetes 2007 Jul;56(7):1761-1772.
12. Farhadi A, Gundlapalli S, Shaikh M, Frantzides C, Harrell L, Kwasny MM, et al. Susceptibility to gut leakiness: a possible mechanism for endotoxaemia in non-alcoholic steatohepatitis. Liver Int 2008 Apr 5.
13. Creely SJ, McTernan PG, Kusminski CM, Fisher M, Da Silva NF, Khanolkar M, et al. Lipopolysaccharide activates an innate immune system response in human adipose tissue in obesity and type 2 diabetes. Am J Physiol Endocrinol Metab 2007 Mar;292(3):E740-747.
14. Ruiz AG, Casafont F, Crespo J, Cayon A, Mayorga M, Estebanez A, et al. Lipopolysaccharide-binding protein plasma levels and liver TNF-alpha gene expression in obese patients: evidence for the potential role of endotoxin in the pathogenesis of non-alcoholic steatohepatitis. Obes Surg 2007 Oct;17(10):1374-1380.
15. Dixon AN, Valsamakis G, Hanif MW, Field A, Boutsiadis A, Harte A, et al. Effect of the orlistat on serum endotoxin lipopolysaccharide and adipocytokines in South Asian individuals with impaired glucose tolerance. Int J Clin Pract 2008 Jul;62(7):1124-1129.
16. Vitseva OI, Tanriverdi K, Tchkonia TT, Kirkland JL, McDonnell ME, Apovian CM, et al. Inducible Toll-like receptor and NF-kappaB regulatory pathway expression in human adipose tissue. Obesity (Silver Spring) 2008 May;16(5):932-937.
17. Ghanim H, Aljada A, Hofmeyer D, Syed T, Mohanty P, Dandona P. Circulating mononuclear cells in the obese are in a proinflammatory state. Circulation 2004 Sep 21;110(12):1564-1571.
18. Wolowczuk I, Verwaerde C, Viltart O, Delanoye A, Delacre M, Pot B, et al. Feeding our immune system: impact on metabolism. Clin Dev Immunol 2008;2008:639803.
19. Mookerjee RP, Stadlbauer V, Lidder S, Wright GA, Hodges SJ, Davies NA, et al. Neutrophil dysfunction in alcoholic hepatitis superimposed on cirrhosis is reversible and predicts the outcome. Hepatology 2007 Sep;46(3):831-840.
20. Sheu WH, Chang TM, Lee WJ, Ou HC, Wu CM, Tseng LN, et al. Effect of Weight Loss on Proinflammatory State of Mononuclear Cells in Obese Women. Obesity (Silver Spring) 2008 May;16(5):1033-1038.

21. Membrez M, Blancher F, Jaquet M, Bibiloni R, Cani PD, Burcelin RG, et al. Gut microbiota modulation with norfloxacin and ampicillin enhances glucose tolerance in mice. Faseb J 2008 Mar 7.
22. Cani PD, Neyrinck AM, Fava F, Knauf C, Burcelin RG, Tuohy KM, et al. Selective increases of bifidobacteria in gut microflora improve high-fat-diet-induced diabetes in mice through a mechanism associated with endotoxaemia. Diabetologia 2007 Nov;50(11):2374-2383.
23. Li Z, Yang S, Lin H, Huang J, Watkins PA, Moser AB, et al. Probiotics and antibodies to TNF inhibit inflammatory activity and improve nonalcoholic fatty liver disease. Hepatology 2003 Feb;37(2):343-350.
24. Shirota M, Aso K, Iwabuchi A. [Studies on intestinal microflora. 1. Its constitution in healthy infants and the effect of oral administration of L. acidophilus strain Shirota]. Nippon Saikingaku Zasshi 1966 May;21(5):274-283.
25. Spanhaak S, Havenaar R, Schaafsma G. The effect of consumption of milk fermented by Lactobacillus casei strain Shirota on the intestinal microflora and immune parameters in humans. Eur J Clin Nutr 1998 Dec;52(12):899-907.
26. Tuohy KM, Pinart-Gilberga M, Jones M, Hoyles L, McCartney AL, Gibson GR. Survivability of a probiotic Lactobacillus casei in the gastrointestinal tract of healthy human volunteers and its impact on the faecal microflora. J Appl Microbiol 2007 Apr;102(4):1026-1032.
27. Asahara T, Nomoto K, Watanuki M, Yokokura T. Antimicrobial activity of intraurethrally administered probiotic Lactobacillus casei in a murine model of Escherichia coli urinary tract infection. Antimicrob Agents Chemother 2001 Jun;45(6):1751-1760.
28. Lee YK, Lim CY, Teng WL, Ouwehand AC, Tuomola EM, Salminen S. Quantitative approach in the study of adhesion of lactic acid bacteria to intestinal cells and their competition with enterobacteria. Appl Environ Microbiol 2000 Sep;66(9):3692-3697.
29. Takeda K, Okumura K. Effects of a fermented milk drink containing Lactobacillus casei strain Shirota on the human NK-cell activity. J Nutr 2007 Mar;137(3 Suppl 2):791S-793S.
30. Stadlbauer V, Mookerjee RP, Hodges S, Wright GA, Davies NA, Jalan R. Effect of probiotic treatment on deranged neutrophil function and cytokine responses in patients with compensated alcoholic cirrhosis. J Hepatol 2008 Jun;48(6):945-951.
31. Matthews DR, Hosker JP, Rudenski AS, Naylor BA, Treacher DF, Turner RC. Homeostasis model assessment: insulin resistance and beta-cell function from fasting plasma glucose and insulin concentrations in man. Diabetologia 1985;28(7):412-419.
32. Stumvoll M, van Haeften T, Fritsche A, Gerich J. Oral glucose tolerance test indexes for insulin sensitivity and secretion based on various availabilities of sampling times. Diabetes Care 2001;24:796-797.
33. Anderwald C, Anderwald-Stadler M, Promintzer M, Prager G, Mandl M, Nowotny P, et al. The Clamp-Like Index: a novel and highly sensitive insulin sensitivity index to calculate hyperinsulinemic clamp glucose infusion rates from oral glucose tolerance tests in nondiabetic subjects. Diabetes Care 2007 Sep;30(9):2374-2380.
34. Jensen CC, Cnop M, Hull RL, Fujimoto WY, Kahn SE, and the American Diabetes Association GENNID Study Group. ß-Cell function is a major contributor to oral glucose tolerance in high-risk relatives of four ethnic groups in the U.S. Diabetes 2002;51:2170-2178.
35. van Hecke MV, Dekker JM, Nijpels G, Moll AC, Heine RJ, Bouter LM, et al. Inflammation and endothelial dysfunction are associated with retinopathy; the Hoorn Study. Diabetologia 2005;48:1300-1306.
36. Hurley JC, Tosolini FA, Louis WJ. Quantitative Limulus lysate assay for endotoxin and the effect of plasma. J Clin Pathol 1991 Oct;44(10):849-854.
