# Supplementary material for: Lactobacillus casei Shirota Supplementation Does Not Restore Gut Microbiota Composition and Gut Barrier in Metabolic Syndrome: A Randomized Pilot Study
Source: PLoS One. 2015 Oct 28;10(10):e0141399. doi: 10.1371/journal.pone.0141399 (PMC4625062; doi:10.1371/journal.pone.0141399)
Supplement: S1 Table — Amplicons were sequenced from the Titanium A adaptor (CCATCTCATCCCTGCGTGTCTCCGAC), followed by a 4 bases key sequence (TCAG) and the 10 bases barcode. The reverse primer was used with the Titanium B adaptor (CCTATCCCCTGTGTGCCTTGGCAGTC), the key sequence and the target specific sequence but without barcode sequence (CCTATCCCCTGTGTGCCTTGGCAGTC TCAG ATTACCGCGGCTGCTGG). (DOCX) [file pone.0141399.s003.docx]

**S1 Table**: **Barcoded primer sequences used in this study**: amplicons were sequenced from the Titanium A adaptor (CCATCTCATCCCTGCGTGTCTCCGAC), followed by a 4 bases key sequence (TCAG) and the 10 bases barcode. The reverse primer was used with the Titanium B adaptor (CCTATCCCCTGTGTGCCTTGGCAGTC), the key sequence and the target specific sequence but without barcode sequence (CCTATCCCCTGTGTGCCTTGGCAGTC TCAG ATTACCGCGGCTGCTGG).

sample ID Barcode ID Barcode lane

1 1 ACGAGTGCGT 1

2 2 ACGCTCGACA 1

3 3 AGACGCACTC 1

4 4 AGCACTGTAG 1

5 5 ATCAGACACG 1

6 6 ATATCGCGAG 1

7 7 CGTGTCTCTA 1

8 8 CTCGCGTGTC 1

9 10 TCTCTATGCG 1

10 11 TGATACGTCT 1

11 13 CATAGTAGTG 1

12 14 CGAGAGATAC 1

13 15 ATACGACGTA 1

14 16 TCACGTACTA 1

15 17 CGTCTAGTAC 1

16 18 TCTACGTAGC 1

17 19 TGTACTACTC 1

18 20 ACGACTACAG 1

19 21 CGTAGACTAG 1

20 22 TACGAGTATG 1

21 23 TACTCTCGTG 1

22 24 TAGAGACGAG 1

23 25 TCGTCGCTCG 1

24 26 ACATACGCGT 1

25 27 ACGCGAGTAT 1

26 28 ACTACTATGT 1

27 29 ACTGTACAGT 1

28 30 AGACTATACT 1

29 31 AGCGTCGTCT 1

30 32 AGTACGCTAT 1

31 41 TAGTGTAGAT 1

32 1 ACGAGTGCGT 2

33 2 ACGCTCGACA 2

34 3 AGACGCACTC 2

35 4 AGCACTGTAG 2

36 5 ATCAGACACG 2

37 6 ATATCGCGAG 2

38 7 CGTGTCTCTA 2

39 8 CTCGCGTGTC 2

40 10 TCTCTATGCG 2

41 11 TGATACGTCT 2

42 13 CATAGTAGTG 2

43 14 CGAGAGATAC 2

44 15 ATACGACGTA 2

45 16 TCACGTACTA 2

46 17 CGTCTAGTAC 2

47 18 TCTACGTAGC 2

48 19 TGTACTACTC 2

49 20 ACGACTACAG 2

50 21 CGTAGACTAG 2

51 22 TACGAGTATG 2

52 23 TACTCTCGTG 2

53 24 TAGAGACGAG 2

54 25 TCGTCGCTCG 2

55 26 ACATACGCGT 2

56 27 ACGCGAGTAT 2

57 28 ACTACTATGT 2

58 29 ACTGTACAGT 2

59 30 AGACTATACT 2
