# Supplementary material for: Lactobacillus casei Shirota Supplementation Does Not Restore Gut Microbiota Composition and Gut Barrier in Metabolic Syndrome: A Randomized Pilot Study
Source: PLoS One. 2015 Oct 28;10(10):e0141399. doi: 10.1371/journal.pone.0141399 (PMC4625062; doi:10.1371/journal.pone.0141399)
Supplement: S2 Table — Median abundance is given for each group. (DOCX) [file pone.0141399.s004.docx]

**S2 Table: Abundance of gut microbiota concerning *Lc*S supplementation.** Median abundance is given for each group.

|  | Healthy controls (n=16) | Standard therapy (n=15) | | *Lc*S (n=13) | |
| --- | --- | --- | --- | --- | --- |
| **Class** |  | base | EOS | base | EOS |
| Bacilli (F) | 0.23% | 0.40% | 0.55% | 0.18% | 0.08% |
| Bacteroida (B) | 55.25% | 43.45% | 36.59% | 40.11% | 41.96% |
| Betaproteobacteria (P) | 0.73% | 0.91% | 0.48% | 0.94% | 0.74% |
| Clostridia (F) | 34.57% | 54.70% | 49.63% | 55.43% | 48.74% |
| Others | 1.04% | 0.68% | 1.32% | 0.66% | 0.38% |
| **Family** |  |  |  |  |  |
| Alcaligenaceae (P) | 0.93% | 1.13% | 1.48% | 0.88% | 1.10% |
| Anaeroplasmataceae (T) | 0.06% | 0.00% | 0.00% | 2.10% | 2.47% |
| Bacteroidaceae (B) | 42.38% | 26.25% | 22.41% | 32.64% | 25.84% |
| Clostridiaceae (F) | 0.48% | 0.79% | 1.13% | 0.42% | 0.22% |
| Enterobacteriaceae (P) | 3.72% | 0.11% | 0.28% | 1.41% | 0.14% |
| Enterococcaceae (F) | 0.02% | 0.01% | 0.02% | 0.04% | 4.82% |
| Erysipelotrichaceae (F) | 0.73% | 0.57% | 1.01% | 0.11% | 0.14% |
| Lachnospiraceae (F) | 17.37% | 27.56% | 24.31% | 31.01% | 26.21% |
| Others | 3.58% | 6.63% | 5.83% | 6.35% | 6.30% |
| Peptostreptococcaceae (F) | 1.48% | 1.55% | 2.23% | 1.00% | 0.41% |
| Porphyromonadaceae (B) | 2.33% | 1.90% | 3.13% | 1.56% | 10.39% |
| Prevotellaceae (B) | 8.80% | 8.20% | 12.33% | 0.00% | 0.00% |
| Rikenellaceae (B) | 0.86% | 1.54% | 1.59% | 0.71% | 0.65% |
| Ruminococcaceae (F) | 12.22% | 15.55% | 17.26% | 15.55% | 16.38% |
| S24-7 (B) | 0.03% | 0.82% | 0.34% | 1.76% | 0.03% |
| Veillonellaceae (F) | 2.38% | 4.01% | 3.82% | 2.40% | 2.28% |

*Lc*S: *Lactobacillus casei* Shirota; B: Bacteroidetes; F: Firmicutes; P: Proteobacteria; T: Tenericutes
